# Assessment of the integrated disease surveillance and response system implementation in health zones at risk for viral hemorrhagic fever outbreaks in North Kivu, Democratic Republic of the Congo, following a major Ebola outbreak, 2021

**DOI:** 10.1186/s12889-024-18642-3

**Published:** 2024-04-24

**Authors:** Ruth Kallay, Gisèle Mbuyi, Carrie Eggers, Soumaila Coulibaly, David Tiga Kangoye, Janvier Kubuya, Gnakub Norbert Soke, Mathias Mossoko, Ditu Kazambu, Alain Magazani, Peter Fonjungo, Richard Luce, Aaron Aruna

**Affiliations:** 1 Division of Global Health Protection, Centers for Disease Control and Prevention, 1600 Clifton Rd NE, Atlanta, GA 30329 USA; 2 National Epidemiology Surveillance Direction, DRC Ministry of Health, Hygiene and Prevention Kinshasa, Kinshasa, Democratic Republic of the Congo; 3 Division of Global Health Protection, Centers for Disease Control and Prevention, Bizzell US, Kinshasa, Democratic Republic of the Congo; 4 North Kivu Provincial Health Direction, DRC Ministry of Health, Hygiene and Prevention, Goma, Democratic Republic of the Congo; 5 Division of Global Health Protection, Centers for Disease Control and Prevention, Kinshasa, Democratic Republic of the Congo; 6 African Field Epidemiology Network, Kinshasa, Democratic Republic of the Congo; 7 Division of Global HIV and Tuberculosis, Centers for Disease Control and Prevention, Kinshasa, Democratic Republic of the Congo

**Keywords:** Integrated disease surveillance and response (IDSR), Electronic integrated disease surveillance and response, Assessment, Surveillance, IDSR implementation, North Kivu, Democratic Republic of the Congo, Disease reporting, Identification, Investigation, Response

## Abstract

**Background:**

The Democratic Republic of the Congo (DRC) experienced its largest Ebola Virus Disease Outbreak in 2018–2020. As a result of the outbreak, significant funding and international support were provided to Eastern DRC to improve disease surveillance. The Integrated Disease Surveillance and Response (IDSR) strategy has been used in the DRC as a framework to strengthen public health surveillance, and full implementation could be critical as the DRC continues to face threats of various epidemic-prone diseases. In 2021, the DRC initiated an IDSR assessment in North Kivu province to assess the capabilities of the public health system to detect and respond to new public health threats.

**Methods:**

The study utilized a mixed-methods design consisting of quantitative and qualitative methods. Quantitative assessment of the performance in IDSR core functions was conducted at multiple levels of the tiered health system through a standardized questionnaire and analysis of health data. Qualitative data were also collected through observations, focus groups and open-ended questions. Data were collected at the North Kivu provincial public health office, five health zones, 66 healthcare facilities, and from community health workers in 15 health areas.

**Results:**

Thirty-six percent of health facilities had no case definition documents and 53% had no blank case reporting forms, limiting identification and reporting. Data completeness and timeliness among health facilities were 53% and 75% overall but varied widely by health zone. While these indicators seemingly improved at the health zone level at 100% and 97% respectively, the health facility data feeding into the reporting structure were inconsistent. The use of electronic Integrated Disease Surveillance and Response is not widely implemented. Rapid response teams were generally available, but functionality was low with lack of guidance documents and long response times.

**Conclusion:**

Support is needed at the lower levels of the public health system and to address specific zones with low performance. Limitations in materials, resources for communication and transportation, and workforce training continue to be challenges. This assessment highlights the need to move from outbreak-focused support and funding to building systems that can improve the long-term functionality of the routine disease surveillance system.

**Supplementary Information:**

The online version contains supplementary material available at 10.1186/s12889-024-18642-3.

## Background

Integrated disease surveillance and response (IDSR) is a disease surveillance framework that aims to improve public health surveillance and response to priority diseases, conditions, and public health events in the African region [1, 2]. The IDSR strategy was developed to respond to infectious disease outbreaks with high death tolls occurring across several African nations in the 1990s [3]. The World Health Organization African Region (WHO-AFRO), in collaboration with ministries of health and their partners, developed the strategy to strengthen surveillance capacity in developing nations in order to detect and respond more quickly to new disease outbreaks [4, 5]. IDSR strategy was adopted by the Democratic Republic of the Congo (DRC) in 2000 and continues to be used in the DRC (DRC Ministry of Public Health: Evaluation du Systeme de Surveillance Epidemiologique dans les Divisions Provinciales de la Sante et dans les Zones de Sante a Risque de la Maladie a Virus Ebola en Republique Democratique du Congo, unpublished; [6]). Its importance is demonstrated in the DRC with the myriad of infectious disease outbreaks that have occurred over the recent years, including the 2018–2020 Ebola virus disease (EVD) outbreak in Eastern DRC, the 2019–2020 Measles outbreak, and the 2019 Coronavirus (COVID-19) Pandemic [7–9].

The third and latest edition of the IDSR guidelines was unveiled in March 2019 and included electronic integrated disease surveillance and response- the application of electronic tools to provide more timely data for surveillance and response [1]. The DRC began implementing electronic surveillance data reporting through District Health Information System 2 (DHIS2) in 2014, however, to date, the DHIS2 rollout has yet to be fully implemented below the health zone level; Microsoft Excel (MS Excel), EpiInfo and EpiData are still used [10]. The DHIS2 system can be used for data input of both aggregated and case-based surveillance data and has been developed for the 21 reportable diseases and conditions in the DRC. The tool allows for the immediate reporting, aggregation, and analysis of disease surveillance data, facilitating the availability of analyses for the use in informed decision making [11]. The implementation of electronic IDSR reporting has been an ongoing process in the DRC but could be critical to preventing future large-scale outbreaks of infectious diseases like the 2018–2020 EVD outbreak [7]. Use of a real-time electronic data reporting system can contribute to improving early detection and notification of outbreaks by increasing the speed of data transmission and improving data quality [12]. These improvements coupled with rapid control measures can reduce illness and death rates within an event and prevent small outbreaks from becoming large-scale protracted outbreaks [13].

In 2016, an IDSR assessment was conducted by the DRC Ministry of Health supported by the Japanese International Cooperation Agency focused on evaluating provinces and health zones in the DRC at risk for an EVD outbreak (DRC Ministry of Public Health: Evaluation du Systeme de Surveillance Epidemiologique dans les Divisions Provinciales de la Sante et dans les Zones de Sante a Risque de la Maladie a Virus Ebola en Republique Democratique du Congo, unpublished). This evaluation concluded that the core functions of identification, notification and investigation were sufficiently performed, but that lack of resources such as trained personnel and equipment, limited functioning of the system (DRC Ministry of Public Health: Evaluation du Systeme de Surveillance Epidemiologique dans les Divisions Provinciales de la Sante et dans les Zones de Sante a Risque de la Maladie a Virus Ebola en Republique Democratique du Congo, unpublished). Response capacity was identified as particularly low (DRC Ministry of Public Health: Evaluation du Systeme de Surveillance Epidemiologique dans les Divisions Provinciales de la Sante et dans les Zones de Sante a Risque de la Maladie a Virus Ebola en Republique Democratique du Congo, unpublished). Among the recommendations an emphasis was put on enhancing the timely sharing of information through electronic IDSR reporting (DRC Ministry of Public Health: Evaluation du Systeme de Surveillance Epidemiologique dans les Divisions Provinciales de la Sante et dans les Zones de Sante a Risque de la Maladie a Virus Ebola en Republique Democratique du Congo, unpublished). A 2018 Joint External Evaluation of the International Health Regulation capacity in the DRC, similarly highlighted the use of electronic systems in surveillance data reporting and emergency response activation as priority areas to be addressed to strengthen the public health system in the DRC [14].

From 2018–2020 the DRC experienced the second largest EVD outbreak worldwide and the largest in DRC history (the 10th outbreak in the DRC), with 3,470 cases and 2,287 deaths, spanning three provinces [15]. The 2018–2020 EVD Outbreak in Eastern DRC led to efforts to re-evaluate the IDSR implementation post-epidemic and assess the ability of the public health system to respond to another public health event. During this outbreak, North Kivu province experienced the majority of EVD cases, and thus, most emergency preparedness and response efforts were concentrated there [15, 16]. In addition, significant funding and international support, including healthcare worker trainings, strengthening lab capacity through procuring equipment and dissemination of infection prevention and control training packages, were provided to the health system in North Kivu province as a result of the EVD outbreak in order to improve disease surveillance and response [16]. It was important to determine the long-term impact of this support in North Kivu with respect to the ability of the system to detect and respond to new outbreaks of infectious diseases, as the DRC remains a hotbed for epidemic prone diseases such as EVD, measles, cholera and emerging infections such as COVID-19 [7–9, 17].

Therefore, an IDSR assessment was planned to assess the capabilities of the routine disease surveillance system and IDSR strategy implementation in North Kivu following this major EVD outbreak. The aim of this study was to evaluate the performance in core and supporting functions of the IDSR system in North Kivu Province Health Zones at risk of an EVD epidemic, to determine the system’s capacity to identify, report, investigate and respond to epidemic prone diseases. Similar evaluations have been conducted in other countries, including Nigeria [12], Uganda [18], and Madagascar [19], as well as in the DRC previously in 2016 (5), and helped inform the current process for evaluating IDSR core functions [20].

## Methods

### IDSR Implementation in the DRC

**Fig. 1:**
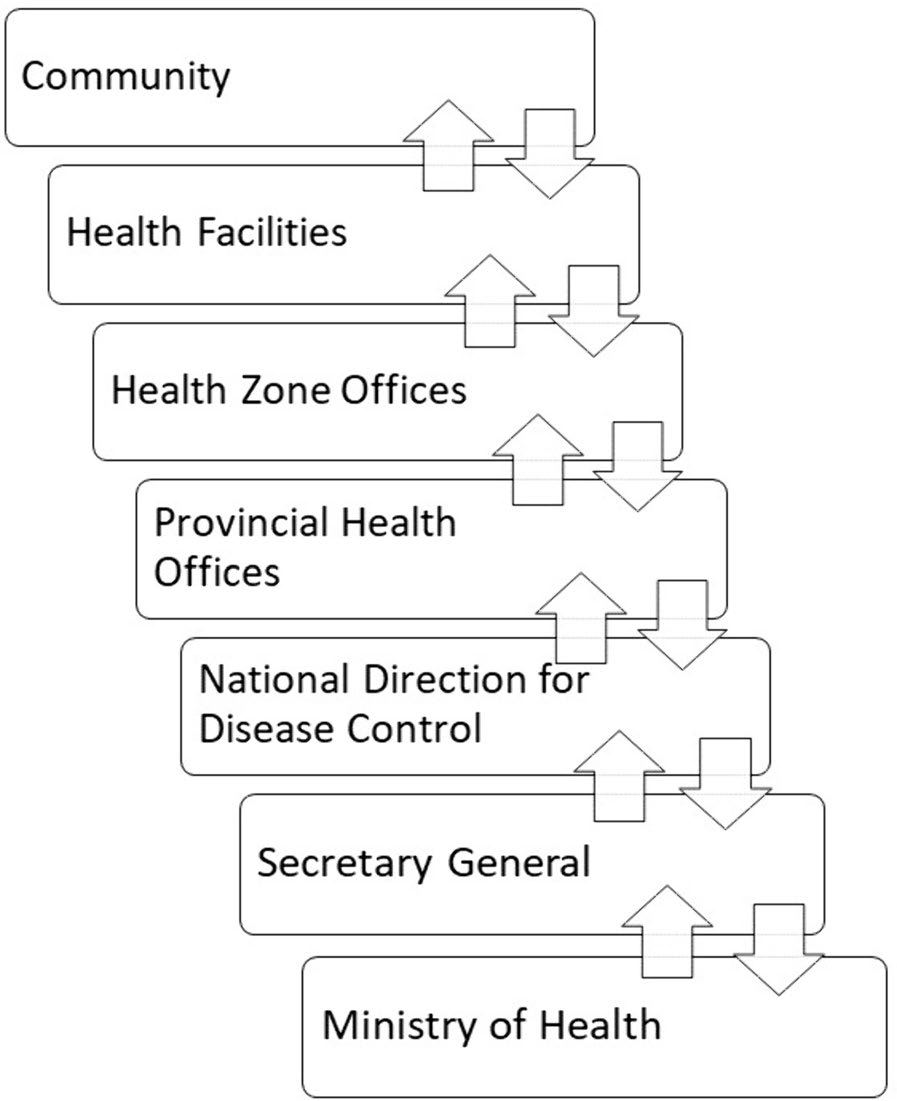
Organization of the flow of surveillance data transmission in the Democratic Republic of the Congo

The information flow of surveillance data in the DRC’s public health system is organized hierarchically, into levels as shown in Fig. 1. At the local level, community outreach and health facilities, serve as the primary sources of disease surveillance data, capturing and recording patient information. In North Kivu, private health clinics are common and frequented by the population. Both public and some private facilities are integrated in the IDSR disease reporting system. These data are typically transmitted to a sentinel health center, where data from all reporting facilities in the health area are collated and reported to the health zone level. Each health zone has a central public health office to collect disease data and supervise surveillance, which links the health facilities to the provincial level. Aggregated data from the health zone level is transmitted to the provincial and national health authorities. IDSR data is reported weekly through this process, with certain conditions requiring immediate reporting or separate additional reporting procedures.

### Survey setting and sampling

**Fig. 2:**
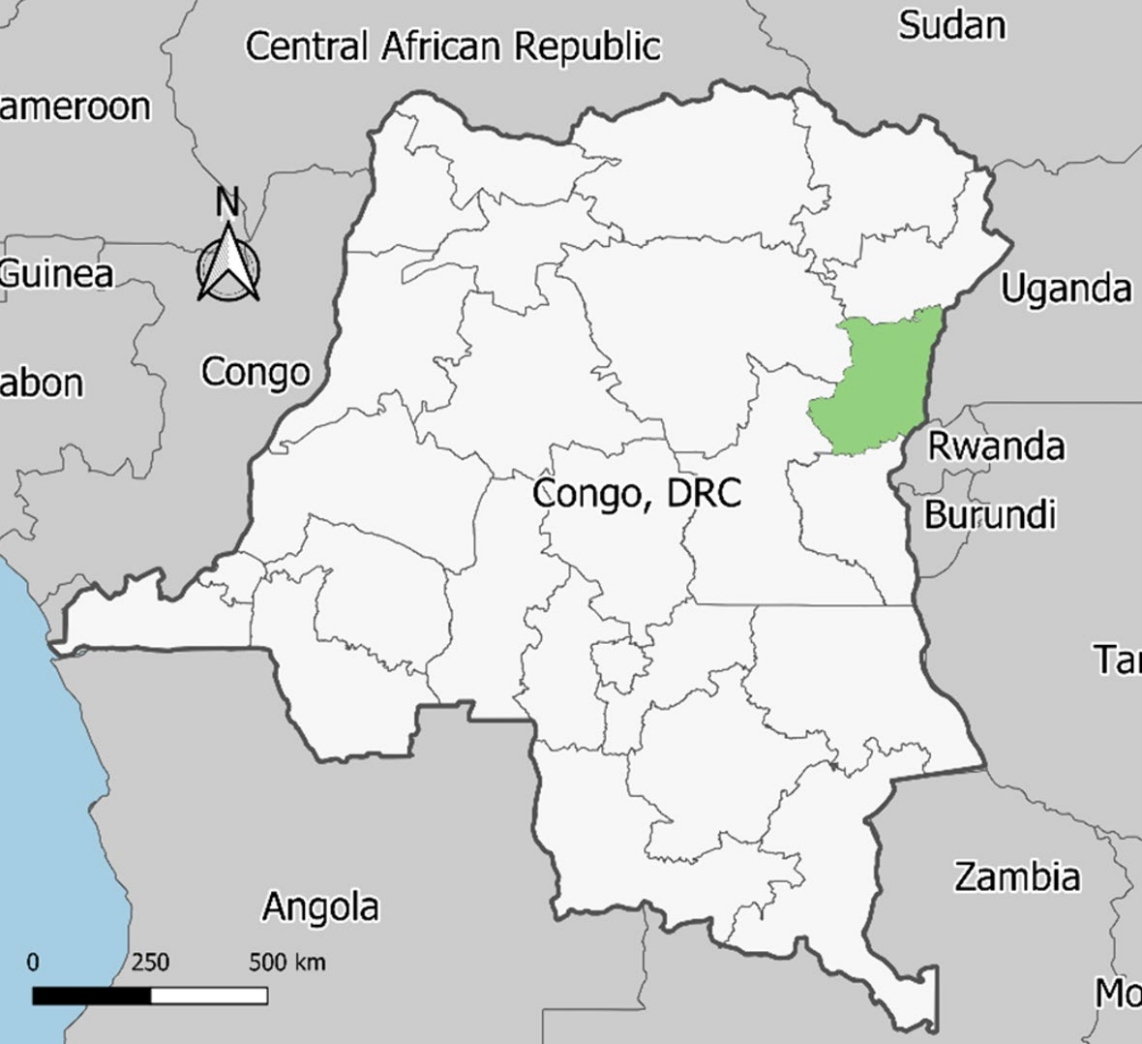
Map of the Democratic Republic of the Congo, North Kivu Province, highlighted

**Fig. 3:**
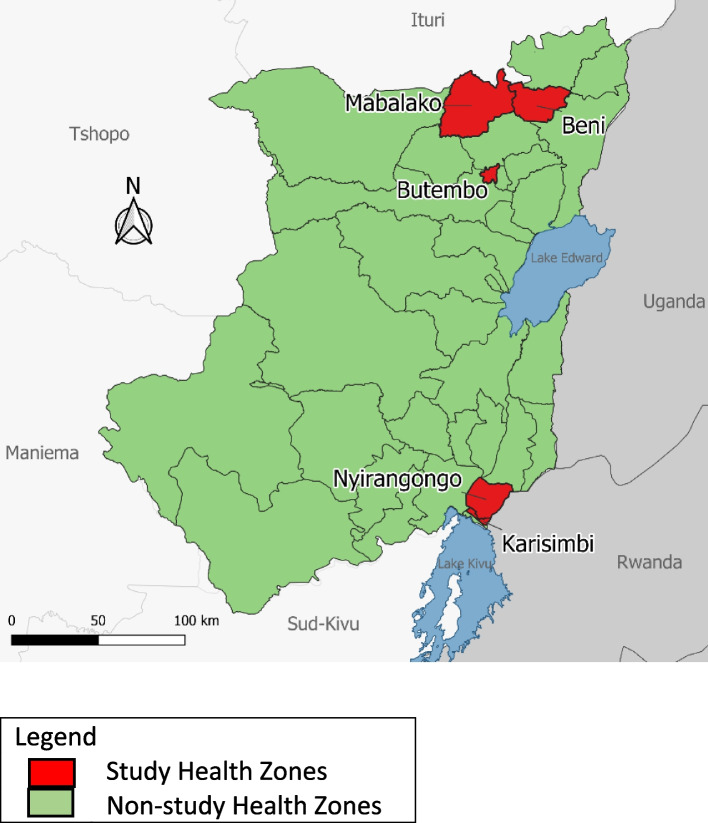
Map of North Kivu Province selected Health Zones, DRC

North Kivu province, shown highlighted in Fig. 2, was selected as the focus for the IDSR assessment due to its prominence during the 2018–2020 EVD outbreak and the risk for future epidemics. North Kivu province is divided into 34 health zones which are further divided into 617 health areas. For this assessment, we included the provincial health office and five of the 34 health zones, shown in Fig. 3. The selection of five health zones for this study was limited due to the widespread security and accessibility issues within this province of the DRC. Additionally, due to the focus on zones that had received increased support during the previous outbreak, the 5 zones were clustered around key areas that had previously had active transmission. The criteria for selection of health zones and health facilities were: localization in a secure area for interviewers to access, already integrated into the IDSR disease reporting system, a mixture of rural to urban settings, high to low performance in disease reporting, and inclusion of areas with a history of EVD cases as well as those without a known history of EVD. The characteristics of selected zones are shown in Table 1.

**Table 1:** Characteristics of selected Health Zones

Health Zone	Urban/Rural	History of EVD cases in 10th outbreak	Proportion of health facilities implementing IDSR
Beni	Urban	Yes	24/306 (8%)
Butembo	Urban	Yes	34/137 (25%)
Karisimbi	Urban	No	63/205 (31%)
Nyiragongo	Rural	Yes	27/163 (17%)
Mabalako	Rural	Yes	71/100 (71%)
**Total**	**-**	**-**	**219/911 (24%)**

Due to security and accessibility issues as well as the lack of a comprehensive list of health facilities in each zone, a random selection of health facilities was not possible, and the listed criteria were used. A total of 66 health facilities were chosen, including the provincial hospital and 65 other health facilities within the five selected health zones. In each health zone, the facilities chosen were selected from at least three different health areas to prevent a narrow geographic focus. The facilities ranged from general reference hospitals, which are usually the most significant health facility within a health zone, to health posts and other small private health clinics in the health areas. The distribution of selected health facilities by the level of the health system and funding source are shown in Table 2.

**Table 2:** Characteristics of participants and health facilities

Characteristic	Number (%)
***Questionnaire Respondents (n* = 72)**	
Provincial Office Level	1 (1%)
Health Zone Office Level	5 (7%)
Health Care Facility Level	66 (92%)
***Qualifications of Questionnaire Respondents (n* = 72)**	
Medical Biologist	1 (1%)
Medical Doctor	2 (3%)
Nurse A1	29 (40%)
Nurse A2	26 (36%)
Nurse A3	4 (6%)
Nurse L3	7 (10%)
Public Health license	3 (4%)
***Healthcare Facility Level (n* = 66)**	
Provincial Hospital	1 (2%)
General Reference Hospital	5 (8%)
Health Center	17 (36%)
Health Post	10 (15%)
Other	33 (50%)
***Funding Source of Facility (n* = 66)**	
Private	32 (48%)
Public	22 (33%)
Religious	12 (18%)
***Type of Medicine Practiced at Facility (n* = 66)**	
Modern	61 (92%)
Traditional or Traditional/Modern	5 (8%)

### Study design

The study utilized a mixed-methods design consisting of quantitative and qualitative methods. Quantitative assessment of the performance in IDSR core functions was conducted at multiple levels of the tiered health system through analysis of health data and a standardized questionnaire administered to health workers who served as surveillance focal points. Qualitative data were also collected through observations, focus groups, and open-ended questions to guide the interpretation of the findings.

### Data collection

The WHO-AFRO’s 3rd edition IDSR guidelines and the Centers for Disease Control and Prevention’s Guidelines for Evaluating Public Health Surveillance Systems were consulted to help design the questions in the quantitative and qualitative data collection tools [1, 21].

For quantitative data, three questionnaires were developed for different levels of the health system (provincial health office, health zone offices, and health facilities). Information was collected on key indicators for 7 IDSR components. This paper will cover the results for the core functions (identification, reporting, investigation, and response) as well as support functions, such as training and resources. The questionnaires consisted of several parts: a walkthrough checklist of the structure to identify available IDSR guidance and reporting materials, a review of the structure’s surveillance data (e.g. patient registers, aggregated IDSR databases, data reporting logs) to evaluate completeness and timeliness of reporting, and interview questions directed to the surveillance focal point and data manager (if the role existed at the structure) to assess capabilities of the facility and perspectives of how to improve the disease surveillance system. The interview portion was primarily quantitative with a small number of qualitative open-ended questions to gauge perspectives.

At each structure visited, a health worker serving as the surveillance focal point was identified as the person responsible for disease data reporting and receiving surveillance data from lower levels, where applicable. At the provincial level, the questionnaire included questions pertaining to all 34 health zones and at the health zone level, all reporting health facilities in the zone.

The questionnaires were developed in Kobo Toolbox and administered by trained interviewers using tablets with the KoboCollect application. The interviewers transcribed the verbal responses into KoboCollect. The interviews were all conducted in French.

The questionnaires were pre-tested in pilot interviews in two health zone offices and six health facilities. Feedback from the interviewers following the pilot led to a revision of the questionnaires prior to the beginning of the assessment.

The interviewers were selected among the DRC Ministry of Health national and North Kivu provincial level surveillance staff and the Field Epidemiology Training Program residents. There were 6 two-member interview teams, with one team assigned to each of the 5 health zones and the sixth team assisting with provincial level data collection. Each team consisted of one FETP resident paired with one Ministry of Health staff member. They received a 3-day training prior to the start of the assessment focused on the protocol for the evaluation and data collection.

Focus group discussions were conducted within all five selected health zones at each of the 15 health areas visited (three per health zone). Groups consisted of 8–12 participants engaged in community health, such as community outreach volunteers and local political and administrative authorities. The focus group portion consisted of 20 open-ended qualitative questions to prompt discussion among the group regarding their knowledge of community surveillance, concerns, and suggestions to improve community-based disease surveillance. One interviewer on the team served as a moderator, while the other interviewer was assigned as a notetaker. All the focus group discussions, except one (14 out of 15 total), were also recorded using an audio recording application on the tablets. For one focus group, the participants declined audio recording, and only written notes were taken. Following the focus group discussions, the interviewer teams were responsible for using the written notes and audio recordings to type up a transcript of the focus group responses in Microsoft Word (MS Word), which was used for analysis.

### Data management and analysis

For the questionnaires, data from Kobo Toolbox were downloaded into a MS Excel spreadsheet, which was cleaned, and quantitative data were analyzed using the Statistical Analysis System (SAS) software package. Descriptive analyses were conducted of available resources and capabilities at each structure as well as data completeness, timeliness, and response and reporting time. Qualitative data from the open-ended questions in the questionnaires were coded in MS Excel and analyzed thematically, then imported into SAS to generate frequencies. Data were compiled based on theme reflecting concerns of participants expressed as existing challenges of the system or areas of need suggested for improvement (Tables 5 and 6). The frequency of themes shown in the results are supported by relevant quotes from participants in Appendices 3 and 4.

Interviewers visually verified and took photos to record availability of documents at each level to guide disease identification, facilitate reporting and guide rapid response. Case definitions and case report forms for each IDSR reportable condition were documented. Reported availability of documents for Malaria included both suspected Malaria and Malaria with positive rapid diagnostic test, although they are reported separately. The test kits were also visually verified by the interviewers. Availability of labs and Rapid Response Teams (RRTs) were based on testimony from surveillance focal points and corroborated at multiple levels. Surveillance focal points were asked during the interview to report on capability of structure staff to collect specimens for lab testing, training received, data reporting methods, and average response time and lab result turnaround time. Reporting logs at the health zone and provincial offices were used to collect data on reported and investigated epidemics.

Data completeness looked at data from all health facilities reporting in each health zone and was measured by the proportion of structures that reported weekly surveillance data for every reportable disease, including diseases or conditions with zero cases out of total structures integrated into the IDSR system, over the 3-month period prior to the data collection (12 total weekly reports expected per facility). Completeness of the patient register was measured by the number of 4 key variables (patient name, date of visit, signs and symptoms and presence/absence of fever) in the patient register of surveyed facilities that had been filled out for the past 20 entries, out of the expected number of data points (80 total for each facility). The total number of facilities in each health zone with a patient register completeness score of 75% or higher are reported. Timeliness looked at all health facilities reporting in each health zone and was measured by the proportion of total structures that submitted weekly IDSR data by the established deadline of submission during the 3 months prior to the assessment out of total structures integrated into IDSR.

The focus group responses were collected from the interviewers in MS Word files and converted to MS Excel spreadsheet. These data were coded inductively, and thematic analysis was conducted. The data were then imported into SAS where frequencies were generated. These issues were analyzed by theme with responses compiled to reflect overall concerns of participants as was done with the open-ended question data.

## Results

The assessment was conducted in North Kivu during January and February of 2021. The health zones assessed are described in Table 1, and the characteristics of participants and facilities are shown in Table 2. The IDSR indicators on which data were collected are shown in Tables 3 and 4 including the verification of documents, databases of IDSR disease data, reporting logs and patient registers. The summary of qualitative data from surveillance focal points is shown in Table 5 and from the focus groups conducted with community members and leaders are shown in Table 6.

**Table 3:** Key IDSR performance indicators at health facility, health zone and provincial levels

		**Health Facilities by Health Zone**					**Facility Total**	**Health Zone Offices**	**Provincial Office**
**Component**	**Indicator**	**Beni**	**Butembo**	**Karisimbi**	**Mabalako**	**Nyiragongo**			
		***N* = 13**	***N* = 13**	***N* = 13**	***N* = 13**	***N* = 13**	***N* = 66**	***N* = 5**	***N* = 1**
		**n (%)**	**n (%)**	**n (%)**	**n (%)**	**n (%)**	**n (%)**	**n(%)**	**n (%)**
Identification	Standard list of reportable diseases	9 (69%)	2 (15%)	3 (23%)	10 (77%)	6 (46%)	31 (47%)	2 (40%)	1 (100%)
	No standard case definitions	11 (85%)	1 (8%)	5 (38%)	4 (31%)	3 (23%)	24 (36%)	1 (20%)	0 (0%)
Reporting	Standard DRC MSP Patient register	12 (92%)	5 (38%)	9 (69%)	9 (69%)	5 (38%)	41 (62%)	-	-
	Complete variables in patient register	60%	74%	74%	93%	83%	Average 77%	-	-
	No blank case reporting forms	8 (62%)	7 (54%)	3 (23%)	11 (85%)	6 (46%)	35 (53%)	1 (20%)	0 (0%)
	*Completeness (denominators listed)*	*24/24 (100%)*	*34/34 (100%)*	*26/63 (41%)*	*20/27 (74%)*	*13/71 (18%)*	*117/219 (53%)*	*34/34 (100%)*	*-*
	*Timeliness (denominators listed)*	*24/24 (100%)*	*24/34 (71%)*	*26/63 (41%)*	*20/27 (74%)*	*71/71 (100%)*	*165/219 (75%)*	*33/34 (97%)*	*-*
Investigation	Capability to collect specimens for lab testing	6 (46%)	9 (69%)	4 (31%)	9 (69%)	4 (31%)	33 (50%)	5 (100%)	1 (100%)
	Test kits to collect specimens	2 (15%)	6 (46%)	4 (31%)	6 (46%)	4 (31%)	22 (33%)	4 (80%)	1 (100%)
	Avg time to receive EVD lab test results < 3 days	8 (62%)	10 (77%)	1 (8%)	13 (100%)	1 (8%)	34 (52%)	4 (80%)	1 (100%)
	Have laboratory	-	-	-	-	-	-	3 (60%)	1 (100%)
	*Suspected outbreaks notified within 24 h (denominators listed)*	*3/3 (100%)*	*3/3 (100%)*	*3/3 (100%)*	*1/1 (100%)*	*3/3 (100%)*	-	*13/13 (100%)*	*3/3 (100%)*
	*Investigated epidemics with lab results (denominators listed)*	*2/3 (67%)*	*3/3 (100%)*	*3/3 (100%)*	*0/1 (0%)*	*3/3 (100%)*	-	*11/13 (85%)*	*3/3 (100%)*
Response	Have RRT	-	-	-	-	-	-	5 (100%)	1 (100%)
	RRT members have SOP	-	-	-	-	-	-	3 (60%)	0 (0%)
	Average RRT response time < 24 h	-	-	-	-	-	-	3 (60%)	0 (0%)
Training	Surveillance focal point trained in IDSR	11 (85%)	9 (69%)	6 (46%)	10 (77%)	7 (54%)	43 (65%)	4 (80%)	1 (100%)

**Table 4:** Methods of IDSR disease data transmission by level

	**Health Facilities by Health Zone**							
**Methods of IDSR disease data transmission**	**Beni**	**Butembo**	**Karisimbi**	**Mabalako**	**Nyiragongo**	**Facility** **Total**	**Health Zone Offices**	**Provincial Office**
	***N* = 13**	***N* = 13**	***N* = 13**	***N* = 13**	***N* = 13**	***N* = 66**	***N* = 5**	***N* = 1**
	**n (%)**	**n (%)**	**n (%)**	**n (%)**	**n (%)**	**n (%)**	**n (%)**	**n (%)**
Paper forms submitted in person	12 (92%)	13 (100%)	11 (85%)	9 (69%)	11 (85%)	57 (86%)	0 (0%)	0 (0%)
SMS or WhatsApp text message	11 (85%)	5 (38%)	11 (85%)	10 (77%)	8 (62%)	45 (68%)	3 (60%)	0 (0%)
Telephone call	9 (69%)	6 (46%)	8 (62%)	2 (15%)	4 (31%)	29 (44%)	0 (0%)	0 (0%)
DHIS2	4 (31%)	0 (0%)	0 (0%)	0 (0%)	0 (0%)	4 (6%)	2 (40%)	0 (0%)
Email	0 (0%)	0 (0%)	0 (0%)	0 (0%)	0 (0%)	0 (0%)	4 (80%)	1 (100%)

**Table 5:** Frequencies of common responses – qualitative data from surveillance focal points

Surveillance Focal Points—Open-ended questions								
	**Health Facilities**					**Total Health Facilities** ***N* = 309** **n (%)**	**Health Zone Offices** ***N* = 32** **n (%)**	**Provincial Office** ***N* = 6** **n (%)**
**Concerns**	**Beni** ***N* = 73** **n (%)**	**Butembo** ***N* = 34** **n (%)**	**Karisimbi** ***N* = 76** **n (%)**	**Mabalako** ***N* = 53** **n (%)**	**Nyiragongo** ***N* = 68** **n (%)**			
Surveillance guides and data collection tools	15 (21%)	10 (29%)	20 (26%)	12 (23%)	14 (21%)	72 (23%)	2 (6%)	2 (33%)
Communication resources	13 (18%)	4 (12%)	19 (25%)	11 (21%)	21 (31%)	68 (22%)	7 (22%)	1 (17%)
Transportation resources	11 (15%)	3 (9%)	15 (20%)	10 (19%)	21 (31%)	60 (19%)	7 (22%)	0 (0%)
Training needs and capacity building	5 (7%)	5 (15%)	11 (14%)	5 (9%)	8 (12%)	36 (12%)	2 (6%)	1 (17%)
Supervision and/or feedback	9 (12%)	7 (21%)	2 (3%)	6 (11%)	0 (0%)	25 (8%)	2 (6%)	0 (0%)
Motivation of personnel	11 (15%)	2 (6%)	6 (8%)	0 (0%)	2 (3%)	21 (7%)	7 (22%)	0 (0%)
Harmonize data reporting systems	0 (0%)	0 (0%)	0 (0%)	0 (0%)	0 (0%)	0 (0%)	0 (0%)	2 (33%)
Availability of internet/Computers	0 (0%)	0 (0%)	0 (0%)	0 (0%)	0 (0%)	0 (0%)	4 (13%)	0 (0%)
Integration of more structures into IDSR	3 (4%)	0 (0%)	0 (0%)	2 (4%)	0 (0%)	5 (3%)	0 (0%)	0 (0%)

**Table 6:** **F**requencies of common responses – qualitative data from focus group discussions

**Community members engaged in health—Focus groups**						
**Concerns**	**Beni** ***N* = 27** **n (%)**	**Butembo** ***N* = 18** **n (%)**	**Karisimbi** ***N* = 29** **n (%)**	**Mabalako** ***N* = 21** **n (%)**	**Nyiragongo** ***N* = 45** **n (%)**	**Total** ***N* = 140** **n (%)**
Need for resources (communication, transport, educational material)	5 (19%)	5 (28%)	17 (59%)	7 (33%)	13 (29%)	47 (34%)
Motivation of personnel	9 (33%)	3 (17%)	7 (24%)	6 (29%)	9 (20%)	34 (24%)
Community resistance	9 (33%)	5 (28%)	2 (7%)	1 (5%)	8 (18%)	25 (18%)
Training needs and capacity building	2 (7%)	3 (17%)	3 (10%)	2 (10%)	5 (11%)	15 (11%)
**Knowledge of community disease surveillance**	**Beni** ***N* = 29** **n (%)**	**Butembo** ***N* = 11** **n (%)**	**Karisimbi** ***N* = 17** **n (%)**	**Mabalako** ***N* = 20** **n (%)**	**Nyiragongo** ***N* = 28** **n (%)**	**Total** ***N* = 105** **n (%)**
Education and community engagement	3 (10%)	3 (27%)	5 (29%)	9 (45%)	12 (43%)	32 (30%)
Disease detection and notification	10 (34%)	4 (36%)	5 (29%)	7 (35%)	4 (14%)	30 (29%)
Referral to structures/promotion of health services	10 (34%)	3 (27%)	4 (24%)	1 (5%)	6 (21%)	24 (23%)

### Identification

**Fig. 4:**
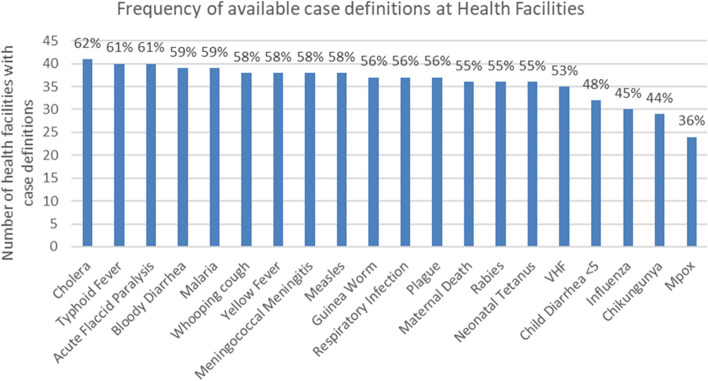
Frequency of available case definitions at Health Facilities

Looking at the availability of disease identification tools shown in Table 2, less than half (47%) of surveyed health facilities had the official list of reportable conditions in the DRC. Only 2 of the 5 health zone offices (40%) had the official list, but it was available at the provincial office level. In more than one-third (36%) of surveyed facilities, no case definitions for any reportable diseases were available. Only one health zone office lacked all case definitions, the other four health zone offices and provincial office all had case definitions for at least one reportable disease. Availability of case definitions at the facilities varied by reportable disease/condition from 36% of facilities having the case definition for monkeypox (mpox) to 62% of facilities having the case definition for cholera, shown in Fig. 4. Within community health workers in the focus groups (Table 6), the most frequent response to the purpose of disease surveillance, at nearly one third of responses (30%) was education and community engagement. The majority of comments on disease surveillance (52%) accurately described community surveillance as either disease detection and notification (29%) or referral of the sick to facilities or health services (23%).

### Reporting

**Fig. 5:**
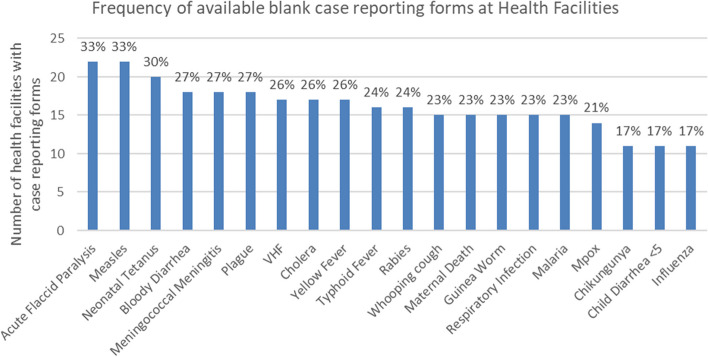
Frequency of available blank case reporting forms at health facilities

A lack of disease case reporting forms was observed, with 53% of the health facilities surveyed having no blank case notification forms available for any reportable diseases. The frequency of available forms by disease or condition varied as shown in Fig. 5, but notably the diseases with available reporting forms did not follow the same pattern as those with available case definitions. 62% of health facilities used the DRC MOH standard patient register to collect patient data. Within the facility patient registers, completeness of key data points was overall 77% complete across all health zones. Data completeness of IDSR reporting from health facilities was 53% overall in the 5 health zones, with Nyiragongo the lowest at 18% and Beni and Butembo the highest at 100%. Timeliness of weekly IDSR data reporting by the health facilities also varied between health zones from 41 to 100%, with Karisimbi health zone being the lowest. However overall, 75% of facilities in the 5 zones consistently reported data on time over a 3-month period, while the timeliness from the health zone level to the provincial level was much higher at 97%. The methods of data transmission used at each level are shown in Table 4, with paper forms being the most common method used by health facilities, and email transmission the prevailing method by health zone offices and the provincial office. Note that several structures used multiple methods of data transmission in parallel. DHIS2 was not a primary data reporting method at any level, and only one health zone (Beni) had any health facilities using DHIS2 for data submission. Qualitative data from the open-ended questions identified lack of authorization from the national level and lack of connectivity as reasons for non-use.

### Investigation

Not all health zones in the province have laboratories, with 3 of the 5 selected zones (60%) having a public or private laboratory. The remaining zones must collect samples and send them to a nearby health zone or provincial lab for testing. 50% of surveyed facilities report having the capability to collect specimens that can be transported for lab testing, however only 33% of surveyed facilities have any test kits available, limiting the facilities that can actually perform sample collection. 52% of health facilities and 80% (4 of 5) of health zone offices report that it takes less than 3 days to receive EVD test results on average, with facilities in health zones Karisimbi and Nyiragongo reporting most frequently lab results take greater than 3 days on average.

Within the health zone and the provincial levels, 100% of suspicions of epidemics in the past year were notified to the superior level within 24 h, and lab results were received by the reporting health zone in 85% of investigated epidemics suggesting the need for additional lab capacity.

### Response

RRTs exist at both the provincial and health zone levels. All of the five health zones surveyed had RRTs, but the provincial health office reported that only 41% of the 34 health zones in the province have functional RRTs. A lack of guidance materials was noted, with 60% (3 of 5) of health zone RRTs having a copy of standard operating procedures (SOP) and the provincial RRT members not having any copies of an SOP. Average response time from notification for the RRT was less than 24 h in 60% of health zones (3 of 5), and response time averaged greater than 24 h for the provincial level RRT.

### Support functions: training and resources

65% of surveyed health facilities had a surveillance focal point that had received IDSR training, while 80% of health zone offices and the provincial office had a surveillance focal point with IDSR training (Table 3). All the levels of the health system evaluated in this assessment from the provincial health office to the health facilities mentioned a lack of adequate resources for their IDSR operations as a frequent concern from the open-ended questions (Table 5). The most common comments overall were: inadequate surveillance guides and data collection tools such as reporting forms and SOPs, insufficient communication tools such as phones and phone credit, and the scarcity of adequate transport. In addition, the need for IDSR training for health personnel was the next most frequent response for health facilities. Increasing the motivation of staff and lack of internet or computers were commonly mentioned at the health zone level. The focus group discussions with community health workers yielded similar common responses, such as the need to supply resources for communication and/or transportation, which were among the most frequent comments. Notably, motivation for personnel and community resistance were also within the topmost frequent comments which differed from the surveillance focal point responses.

## Discussion

The findings of this study have allowed us to assess the IDSR system in selected health zones in North Kivu at risk of a viral hemorrhagic fever outbreak for their performance in core and supporting functions. The core functions of identification, reporting, investigation and response to epidemic prone diseases will be covered here as well as findings related to the support functions of training and resources.

### Identification

The lack of surveillance guidance materials at the health facility and health zone levels is a clear weakness for effecting standardized disease identification. Without a complete list of reportable diseases or standard case definitions, ensuring that healthcare workers at the primary care level are properly identifying suspect cases presents a challenge for many facilities. There is little change from the last IDSR assessment in 2016 in this respect. The availability of the standard list of reportable diseases and conditions in this assessment (47%) is lower than the 65% and 56% availability reported previously in general reference hospitals and health centers respectively (DRC Ministry of Public Health: Evaluation du Systeme de Surveillance Epidemiologique dans les Divisions Provinciales de la Sante et dans les Zones de Sante a Risque de la Maladie a Virus Ebola en Republique Democratique du Congo, unpublished). The availability of case definitions (64%) is slightly higher compared to the previously reported 61% of general reference hospital and 60% of health centers (DRC Ministry of Public Health: Evaluation du Systeme de Surveillance Epidemiologique dans les Divisions Provinciales de la Sante et dans les Zones de Sante a Risque de la Maladie a Virus Ebola en Republique Democratique du Congo, unpublished). The availability of other resources likely impacts these indicators, as the lack of computers and means of photocopying have been noted as challenges. Additional investment in providing guidance materials to all levels and developing a sustainable system to distribute updated identification guides is recommended. Among community health workers, the large number of responses regarding community surveillance that described it as health education or community engagement suggest that additional training of community health workers is needed. While these activities have a role in the public health system, the focus on detecting and reporting suspect cases should be clear to those responsible for conducting surveillance within the community.

### Reporting completeness and timeliness

The vast difference in health facility data completeness by health zone with some zones at 100%, suggests that efforts to improve reporting completeness need to be directed at lower levels of the health system and targeted to zones with low performance rather than sweeping efforts. Country directives focused on providing support to improve reporting at the higher levels of the health system appears to have led to the health zone and the provincial offices ensuring 100% completeness of data reported up for all diseases, while facilities in certain zones, which play the role of primary sources of surveillance data continue to feed incomplete disease data into those aggregated numbers. Similar to data completeness, the variability of timeliness of weekly data reporting coupled with the higher timeliness from the health zones likely means weekly data reports sent by the health zones are lacking data from facilities when submitted and disease reporting at the higher levels likely lags behind actual incidence. Despite seeming improvements in disease reporting indicators at higher levels of the health system, disease incidence is likely to be underreported in IDSR surveillance. Results of a previous review of IDSR disease surveillance data in the DRC show that discrepancies between IDSR reported morbidity and actual morbidity are common among the weekly reported diseases [22]. Availability of standard patient registers and case reporting forms at all facilities may help facilitate improvements in completeness. As well, increased supervision and feedback is needed to support the health facility level to correct issues in zones with lower performance to improve both completeness and timeliness.

The low use of DHIS2 for electronic data reporting at each level, shows a lack of improved capacity since the 2018 Joint External Evaluation of the International Health Regulation capacity, in which the DRC scored a 1, the lowest score meaning no capacity, for the use of electronic systems in surveillance data reporting [14]. From a geographic perspective, the country is vast, with 519 health zones and 10,067 health areas, making efforts to provide equipment and electronic surveillance training down to the health area level countrywide challenging and costly. Significant investments will be needed for additional equipment and training to improve functionality of electronic disease reporting but will be key for improving data timeliness, particularly where many structures are still reliant on in-person transport of paper forms. These efforts should be targeted first at the provincial and health zone levels, where the tools are available but not consistently used, before continuing implementation down to the health facility level. Currently, traditional reporting channels continue to be used in parallel, increasing the workload of surveillance staff and likely contributing to low timeliness.

### Investigation

The lab capacity in North Kivu still has some room for improvement, particularly in ensuring adequate resources for testing and building capacity to collect samples. While the DRC has invested in additional trainings to improve lab capabilities in Goma since the end of the 2018–2020 EVD outbreak, the ability of health facilities to collect specimens for testing is still low [23]. For suspect cases requiring lab confirmation, the majority of facilities need to rely on the patient to visit another referral facility which can collect specimens or the RRTs from higher levels to respond and collect specimens. Both options add to the time needed for confirmation of cases, introduce additional risk of losing the patient to follow-up, and limit early disease detection. Additional training and availability of testing kits at health facilities and within RRTs are needed, as well as improving transportation resources to reduce time to investigate events and receive lab test results.

### Response

The functionality of the RRTs needs to improve, which could be linked to the high turnover of trained personnel and the lack of standardization of the RRTs in accordance with the guidelines of the 3rd edition of the IDSR. The low functionality of RRTs was previously identified in the 2016 IDSR evaluation in the DRC, as less than one-third (31%) of provinces assessed and roughly one in ten (9.7%) health zones had functioning RRTs (DRC Ministry of Public Health: Evaluation du Systeme de Surveillance Epidemiologique dans les Divisions Provinciales de la Sante et dans les Zones de Sante a Risque de la Maladie a Virus Ebola en Republique Democratique du Congo, unpublished). While availability of RRTs in North Kivu and the selected zones is high, at both levels the teams face challenges responding to alerts within 24 h due to logistical, operational, and financial constraints. Recommended priority actions of the 2018 Joint External Evaluation included establishing emergency response teams at all levels of the health system and the development and dissemination of Standard Operating Procedures for public health emergencies [14]. Within North Kivu, the focus for improving response functioning should be on dissemination of SOPs to all RRTs, additional training, and ensuring that RRTs have adequate resources for faster response times.

### Support functions

The implications of inadequate training are relevant to each of the IDSR core functions. Training efforts should be directed towards lower levels of the health system particularly health facility surveillance focal points and community health workers. Training should be on the 3rd edition of IDSR which includes electronic disease reporting, and a sustainable system for continued training should be established due to high turnover of healthcare workers in the DRC.

For disease reporting and investigation to improve, particularly in more remote areas, the challenges of communicating quickly and having means of transportation within each health zone will need to be addressed. In the context of the DRC, outbreaks often occur in remote areas with poor road infrastructure, and mobility often requires a combination of different types of transportation (car, motorbike, canoe, etc.). Increased availability of resources should be directed at lower levels of the health zone where early disease detection is paramount. Zones with low performance and high risk of disease should be prioritized for support to better target weak points as system improvements will be costly. Dissemination of guidance materials should be ubiquitous across the health system however, to ensure standardization of identification and reporting, with means provided to reproduce documents as needed.

Finally, one additional challenge noted among the qualitative responses on healthcare worker perceptions is the need for more health facilities, particularly private clinics to be integrated into the IDSR system. This is supported by the results on proportions of IDSR integrated health facilities by health zone in Table 1. For a region with such a high volume of private clinics, health zones need to strive for integration of all health facilities into the system and to provide surveillance training in order to improve disease detection and reporting.

### Limitations

The sampling of health zones and health facilities was not random, which limits the generalizability and applicability of the findings to the selected zones of North Kivu province. The geographic scope of this assessment was also limited due to security and accessibility issues present in many of the health zones in North Kivu, in order to protect the interviewers, as violence is an ongoing threat in many areas. Also, some health zones identified as lower performing health zones in the province could only be reached by helicopter or several days of dangerous overland transportation and thus were not assessed. North Kivu province, which is a communicable disease hotspot, shares common borders with Uganda and Rwanda. The points of entry in this province are among the most frequented countrywide. However, surveillance activities at points of entry were not specifically assessed. It is also possible that repeated and prolonged disease outbreaks have made disease surveillance in North Kivu more effective than in other provinces, in addition to the significant Ebola related support they have received compared to other provinces.

## Conclusion

The results of this current assessment highlight the IDSR functionality following a major Ebola outbreak and bring into question the lasting effects of acute support provided in an outbreak response. The limited improvements observed in this assessment from previous assessments suggest that the immediate support provided in response to the 2018–2020 Ebola outbreak did not result in significant, sustained improvements to the routine surveillance system in North Kivu. A comprehensive national surveillance strategy is needed to develop surveillance capacity beyond what is implemented in outbreak response. The risk of future public health events in this region is high, and the limitations preventing effective disease surveillance have persisted for years based on past evaluations (DRC Ministry of Public Health: Evaluation du Systeme de Surveillance Epidemiologique dans les Divisions Provinciales de la Sante et dans les Zones de Sante a Risque de la Maladie a Virus Ebola en Republique Democratique du Congo, unpublished; [14]). In the context of the COVID-19 pandemic and the increasing frequency of Ebola outbreaks, the current pace at which infectious diseases emerge and re-emerge make it critical for the DRC government to set IDSR as a top health security priority with adequate domestic budget lines. It is of utmost importance to coordinate partners’ support to the Ministry of Health technically and financially, to ensure the sustainability of progress made over time.

### Supplementary Information

**Supplementary Material 1.**

**Supplementary Material 2.**

**Supplementary Material 3.**

**Supplementary Material 4.**

**Supplementary Material 5.**

## Data Availability

The de-identified dataset is available upon reasonable request to the corresponding author.

## Abbreviations

COVID-19: Coronavirus Disease 2019
DHIS2: District Health Information Software 2
DRC: Democratic Republic of the Congo
EVD: Ebola Virus Disease
IDSR: Integrated Disease Surveillance and Response
MS Excel: Microsoft Excel
MS Word: Microsoft Word
RRT: Rapid Response Teams
SAS: Statistical Analysis System
SOP: Standard Operating Procedures
WHO-AFRO: World Health Organization African Region
