# Supplementary material for: Assessment of the integrated disease surveillance and response system implementation in health zones at risk for viral hemorrhagic fever outbreaks in North Kivu, Democratic Republic of the Congo, following a major Ebola outbreak, 2021
Source: BMC Public Health. 2024 Apr 24;24:1150. doi: 10.1186/s12889-024-18642-3 (PMC11044341; doi:10.1186/s12889-024-18642-3)
Supplement: Supplementary file 1 — Supplementary Material 1. [file 12889_2024_18642_MOESM1_ESM.docx]

**Appendix** **1.** **Priority reportable diseases in DRC**

1. Viral hemorrhagic fever
2. Chikungunya
3. Cholera
4. Whooping cough
5. Maternal deaths
6. Diarrhea and dehydration in children < 5
7. Bloody diarrhea
8. Dracunculiasis (Guinea worm)
9. Yellow fever
10. Typhoid fever
11. Influenza
12. Acute respiratory infection
13. Meningococcal meningitis
14. Mpox
15. Malaria
16. Rapid diagnostic test positive malaria (reported separately from Malaria)
17. Plague
18. Acute flaccid paralysis
19. Rabies
20. Measles
21. Neonatal tetanus
