# Supplementary material for: Assessment of the integrated disease surveillance and response system implementation in health zones at risk for viral hemorrhagic fever outbreaks in North Kivu, Democratic Republic of the Congo, following a major Ebola outbreak, 2021
Source: BMC Public Health. 2024 Apr 24;24:1150. doi: 10.1186/s12889-024-18642-3 (PMC11044341; doi:10.1186/s12889-024-18642-3)
Supplement: Supplementary file 2 — Supplementary Material 2. [file 12889_2024_18642_MOESM2_ESM.docx]

**Appendix 2. Definitions of IDSR indicators**

| **Component** | **Indicator** | **Numerator** | **Denominator** |
| --- | --- | --- | --- |
| Identification | Standard list of reportable diseases | Number of surveyed health structures with the MSP standard list of reportable diseases | Total number of surveyed health structures |
|  | No standard case definitions | Number of surveyed health structures that did not have MSP standard case definitions for any reportable disease or condition | Total number of surveyed health structures |
| Reporting | Standard DRC MSP Patient register | Number of surveyed health facilities with a standard MSP patient register | Total number of surveyed health facilities |
|  | ≥75% complete patient register | Number of surveyed health facilities that had 75% or more of 4 key variables (patient name, date of visit, signs and symptoms and presence/absence of fever) in the patient register that had been filled out for the past 20 entries | Total number of surveyed health facilities |
|  | No blank case reporting forms | Number of surveyed health structures that did not have blank case reporting forms for any reportable disease or condition | Total number of surveyed health structures |
|  | *Completeness (denominators listed)* | Number of health structures that the health zone/provincial office reported receiving weekly surveillance data from that included data for every reportable disease and condition, including those with zero cases over the 3-month period prior to the data collection (12 complete weekly reports expected per facility) | Total number of health structures integrated into the IDSR system in each health zone |
|  | *Timeliness (denominators listed)* | Number of health structures that the health zone/provincial office reported receiving weekly IDSR data for by the established deadline of submission consistently during the 3 months prior to the data collection (12 on time weekly reports expected per facility) | Total number of health structures integrated into the IDSR system in each health zone |
| Investigation | Capability to collect specimens for lab testing | Number of health structures where the surveillance focal point self-reported that structure staff have the capability to collect specimen for lab testing for any reportable disease or condition | Total number of surveyed health structures |
|  | Test kits to collect specimens | Number of health structures that were able to produce at the time of interview at least one test kit to collect specimen for lab testing for any reportable disease or condition | Total number of surveyed health structures |
|  | Avg time to receive EVD lab test results <3 days | Number of health structures where the surveillance focal point self-reported that the average time it took to receive EVD RT-PCR test results from the time of collection was less than 3 days | Total number of surveyed health structures |
|  | Have laboratory | Number of health zones with a private or public laboratory able to test specimens | Total number of surveyed health zones/provincial offices |
|  | *Suspected outbreaks notified within 24 hours (denominators listed)* | *Number of suspected outbreaks notified to the next level of the health system within 24 hours over the past 12 months* | *Number of total suspected outbreaks reported in the health zone in the past 12 months* |
|  | *Investigated epidemics with lab results (denominators listed)* | *Number of suspected outbreaks reported with lab results over the past 12 months* | *Number of total suspected outbreaks reported in the health zone in the past 12 months* |
| Response | Have RRT | Number of health zone/provincial offices with a functioning rapid response team | Total number of health zone/provincial offices |
|  | RRT members have SOP | Number of health zone/provincial offices with a rapid response team where the members have a standard operating procedure | Total number of health zone/provincial offices |
|  | Average RRT response time <24 hours | Number of health zone/provincial offices with a rapid response team that has an average response time of less than 24 hours to respond to alerts of suspected disease | Total number of health zone/provincial offices |
| Training | Surveillance focal point trained in IDSR | Number of health structures where the surveillance focal point self-reported to have received training in IDSR | Total number of surveyed health structures |
