# Supplementary material for: Assessment of the integrated disease surveillance and response system implementation in health zones at risk for viral hemorrhagic fever outbreaks in North Kivu, Democratic Republic of the Congo, following a major Ebola outbreak, 2021
Source: BMC Public Health. 2024 Apr 24;24:1150. doi: 10.1186/s12889-024-18642-3 (PMC11044341; doi:10.1186/s12889-024-18642-3)
Supplement: Supplementary file 3 — Supplementary Material 3. [file 12889_2024_18642_MOESM3_ESM.docx]

**Appendix 3 . Quotes of responses to open-ended questions from surveillance focal points**

| **Concerns** | **Quotes** |
| --- | --- |
| Surveillance guides and data collection tools | "Make collection tools available (list of diseases, list of community and standard case definitions)" |
| Communication resources | "That the authorities make available a telephone and communication credit" |
| Transportation resources | "Provision of a means of transport to support the health facility" |
| Training needs and capacity building | "Train providers in integrated disease surveillance and response" |
| Supervision and/or feedback | "That the health zone office increase the frequency of follow-up and supervision" |
| Motivation of personnel | "That the state improve the social conditions of healthcare providers in health facilities" |
| Harmonize data reporting systems | "Harmonization of databases between the 2 directions" |
| Availability of internet/Computers | "Lack of funding for internet" |
| Integration of more structures into IDSR | "Lack of involvement of private structures in the surveillance of notifiable diseases" |
