# Supplementary material for: Assessment of the integrated disease surveillance and response system implementation in health zones at risk for viral hemorrhagic fever outbreaks in North Kivu, Democratic Republic of the Congo, following a major Ebola outbreak, 2021
Source: BMC Public Health. 2024 Apr 24;24:1150. doi: 10.1186/s12889-024-18642-3 (PMC11044341; doi:10.1186/s12889-024-18642-3)
Supplement: Supplementary file 4 — Supplementary Material 4. [file 12889_2024_18642_MOESM4_ESM.docx]

**Appendix 4 . Quotes of focus group discussion responses from community members**

| **Concerns** | **Quotes** |
| --- | --- |
| Need for resources (communication, transport, educational material) | "When there is a serious illness, there is no telephone to communicate with [the head nurse] or a means of transport" |
| Motivation of personnel | "Let the RECOs be motivated with a little money at the end of each month" |
| Community resistance | "the population is afraid to go to the health center after awareness-raising; there is fear of being labelled with Ebola and Covid-19" |
| Training needs and capacity building | "train RECOs who have never received training, and retrain those who have" |
| **Knowledge of community disease surveillance** | **Quotes** |
| Education and community engagement | "Home visits to teach people about protection and prevention of various diseases in the community." |
| Disease detection and notification | "Home visits to identify diseases in the community, since each RECO is responsible for 50 households and must report community health problems" |
| Referral to structures/promotion of health services | "When we see a sick person in the community, we must educate them to go to the health center" |
