# Supplementary material for: Assessment of the integrated disease surveillance and response system implementation in health zones at risk for viral hemorrhagic fever outbreaks in North Kivu, Democratic Republic of the Congo, following a major Ebola outbreak, 2021
Source: BMC Public Health. 2024 Apr 24;24:1150. doi: 10.1186/s12889-024-18642-3 (PMC11044341; doi:10.1186/s12889-024-18642-3)
Supplement: Supplementary file 5 — Supplementary Material 5. [file 12889_2024_18642_MOESM5_ESM.docx]

**Appendix 5. Focus Group Discussion Questions**

FR : Partie I – 25 min

EN: Part I - 25 min

- FR : Que savez-vous de la surveillance des maladies dans votre communauté ?
- EN: What do you know about disease surveillance in your community?
- FR : Sur la base de ce que vous savez, est-ce utile ?
- EN: Based on your knowledge, is disease surveillance useful?
- FR : Expliquez pourquoi ou pourquoi pas ?
- EN: Explain why or why not?
- FR : Quels sont les maladies dans la communauté qui vous préoccupent le plus ?
- EN: What diseases in the community are you most concerned about?
- FR : Quelles sont les cinq maladies ou affections les plus courantes que vous avez observées dans votre Aire de Santé au cours des six derniers mois ?
- EN: What are the five most common diseases or conditions you have observed in your Health Area over the last six months?
- FR : Quel type d'activités/travail de surveillance avez-vous effectué dans votre Aire de Santé ? Veuillez énumérer toutes les activités.
- EN: What type of surveillance activities/monitoring work have you carried out in your Health Area? Please list all activities.

FR : Partie II – 25 min

EN: Part II - 25 min

- FR : Quel type d'événements ou de maladies recherchez-vous parmi les membres de la communauté dans le cadre de votre travail de surveillance ?
- EN: What type of events or illnesses do you look for among community members as part of your surveillance work?
- FR : À quel moment intervenez-vous ?
- EN: When do you intervene?
- FR : Quand avez-vous communiqué pour la dernière fois des informations à l'Infirmier Titulaire ?
- EN: When did you last communicate information to the Head Nurse (of the Health Area)?
- FR : Quoi avez-vous signalé ?
- EN: What did you report?
- FR : Avez-vous utilisé un téléphone ou un autre moyen de communication ?
- EN: Did you use a telephone or other means of communication?
- FR : À quand remonte la dernière fois que l’IT vous a fourni un retour d'information sur vos activités de surveillance ? Comment vous a-t-il fourni ces informations ?
- EN: When was the last time the Head Nurse provided you with feedback on your monitoring activities? How did they provide you with this information?

FR : Partie III – 25 min

EN: Part III - 25 min

- FR : De quoi avez-vous besoin pour effectuer une surveillance dans votre communauté ?
- EN: What do you need to carry out surveillance in your community?
- FR : Selon vous, quelles sont les principales difficultés pour identifier et déclarer les maladies dans la communauté ?
- EN: In your opinion, what are the main difficulties in identifying and reporting diseases in the community?
- FR : Avez-vous reçu une formation pour mener des activités de surveillance dans l'aire de Santé ?
- EN: Have you received training to carry out surveillance activities in the health area?
- FR : Si oui, quand avez-vous reçu cette formation pour la dernière fois et qui l'a dispensée ?
- EN: If so, when was the last time you received training, and who provided it?
- FR : Si non, quel type de formation ou de matériel vous serait utile ?
- EN: If not, what type of training or materials would be useful to you?
- FR : Quelles suggestions pouvez-vous faire pour améliorer la surveillance des maladies dans votre Aire de Santé ?
- EN: What suggestions can you make to improve disease surveillance in your Health Area?

FR : Remarques complémentaires – 5-10 minute

EN: Additional comments - 5-10 minutes
